# The Protective Effect of Melatonin on Neural Stem Cell against LPS-Induced Inflammation

**DOI:** 10.1155/2015/854359

**Published:** 2015-02-01

**Authors:** Juhyun Song, So Mang Kang, Kyoung Min Lee, Jong Eun Lee

**Affiliations:** ^1^Department of Anatomy, Yonsei University College of Medicine, Brain Korea 21 Project for Medical Science, 50 Yonsei-ro, Seodaemun-gu, Seoul 120-752, Republic of Korea; ^2^BK21 Plus Project for Medical Sciences and Brain Research Institute, Yonsei University College of Medicine, Seoul 120-752, Republic of Korea; ^3^Department of Neurology, Seoul National University College of Medicine, Seoul 151-742, Republic of Korea

## Abstract

Stem cell therapy for tissue regeneration has several limitations in the fact that transplanted cells could not survive for a long time. For solving these limitations, many studies have focused on the antioxidants to increase survival rate of neural stem cells (NSCs). Melatonin, an antioxidant synthesized in the pineal gland, plays multiple roles in various physiological mechanisms. Melatonin exerts neuroprotective effects in the central nervous system. To determine the effect of melatonin on NSCs which is in LPS-induced inflammatory stress state, we first investigated nitric oxide (NO) production and cytotoxicity using Griess reagent assays, LDH assay, and neurosphere counting. Also, we investigated the effect of melatonin on NSCs by measuring the mRNA levels of SOX2, TLX, and FGFR-2. In addition, western blot analyses were performed to examine the activation of PI3K/Akt/Nrf2 signaling in LPS-treated NSCs. In the present study, we suggested that melatonin inhibits NO production and protects NSCs against LPS-induced inflammatory stress. In addition, melatonin promoted the expression of SOX2 and activated the PI3K/Akt/Nrf2 signaling under LPS-induced inflammation condition. Based on our results, we conclude that melatonin may be an important factor for the survival and proliferation of NSCs in neuroinflammatory diseases.

## 1. Introduction

Melatonin is a well-known free radical scavenger, antioxidant [1, 2], and antiapoptotic agent [3, 4]. Circulating melatonin is synthesized in the pineal gland as well as in peripheral tissues and is secreted at high levels in a circadian manner [5]. Melatonin has a variety of important physiological functions, including circadian rhythm regulation as well as visual, reproductive, cerebrovascular, neuroendocrine, and neuroimmunological actions [6, 7]. Melatonin exerts neuroprotective effects in many pathological conditions of the central nervous system (CNS) including Parkinson's disease, Alzheimer's disease, and ischemic brain injury [8, 9]. Recently, it has been reported that melatonin influences cell growth and differentiation of neural stem cells (NSCs) [10, 11]. NSCs are characterized as self-renewing, immature, undifferentiated, and multipotent, indicating that they can differentiate into neurons, astrocytes, and oligodendrocytes [12]. Lately, NSCs have been known as the therapeutic target for neurodegenerative disease. However, several problems should be solved for NSC's clinical application [13, 14]. NSC's survival and proliferation ability are important for increasing the therapeutic potential of NSCs in injured tissue [15, 16]. The effects and mechanism of melatonin on NSC proliferation, apoptosis, and differentiation have been evaluated. However, its mechanism in neuroinflammation is currently unclear. Neuroinflammatory responses result in synaptic impairment, neuronal death, and the exacerbation of several disease pathologies within the brain [17, 18]. An excessive inflammatory response results in severe neurodegenerative diseases [19]. In neuroinflammation state, damaged neurons can be repaired by NSCs [20]. Therefore, the survival, self-renewal, proliferation, and differentiation of NSCs have been emphasized in inflammatory environment [21]. Melatonin protects brain injury against LPS-induced inflammatory condition* in vivo* [22] and regulates antioxidant genes of LPS-stimulated macrophages* in vitro* [23]. In the present study, we investigated the role of melatonin in NSCs during LPS-induced inflammation. Nitric oxide (NO) is an inflammatory molecule [24–26]. NO causes neuronal apoptosis by inhibiting neuronal respiration, which increases glutamate release and results in NMDA receptor-mediated excitotoxic cell death [27]. High NO levels exert their toxic effects through multiple mechanisms including lipid peroxidation, mitochondrial damage, protein nitration and oxidation, depletion of antioxidant reserves, modulation of various signaling pathways, and DNA damage [25, 28, 29]. NO can also induce apoptosis in a variety of cultured cell types, including neurons [30–32], and contribute to the death of neurons in CNS diseases such as ischemic stroke [33] and Alzheimer's disease [34]. In addition, NO is involved in the determination of neural precursor cell (NPC) fate [35] and NSC proliferation [36]. Melatonin suppresses NO production through various mechanisms [37]. Here, we confirmed the inhibitory effect of melatonin on NO production of NSCs against LPS-induced inflammatory stress. Melatonin also influences the proliferation and differentiation activity of NSCs [10]. The transcription factor SRY- (sex-determining region-) box 2 (SOX2) is an important functional marker of NPCs and plays a critical role in self-renewal and neuronal differentiation [38]. NPCs require SOX2 at an early stage of differentiation, promoting dorsal root ganglia (DRG) expression of* NGN1* and* Mash1* [39, 40]. SOX2 regulates important functions in NSCs of the CNS as well as in a variety of other tissue-specific stem/progenitor cells [41]. Orphan nuclear receptor TLX is an essential transcriptional regulator of NSCs maintenance and self-renewal in the adult brain [42]. Fibroblast growth factor receptor-2 (FGFR-2) promotes self-renewal of radial glial cells, increasing neuron production [43, 44], and is associated with the proliferation of embryonic stem cells [44]. Also, FGFR-2 regulates neurogenesis and the number of proliferative cells [45]. Therefore, we investigated whether or not melatonin influences SOX2, TLX, and FGFR-2 expression as crucial factors of NSC proliferation, self-renewal, and survival. Nuclear factor-erythroid 2-related factor 2 (Nrf2) controls the expression of diverse protective genes in response to oxidative stress [46]. Nrf2 induces a cellular rescue pathway that protects against LPS-induced inflammatory stress [47]. Nrf2 enhances cytoprotection in the presence of active phosphatidylinositol 3-kinase (PI3K)/Akt signaling [48]. Melatonin increases the mRNA and protein levels of antioxidant enzymes via Nrf2 activation [49, 50]. In the present study, we examined whether melatonin regulates Nrf2 activation in LPS-treated NSCs. Our results suggest the possibility of melatonin as a regulator of NSC's survival and proliferation for the treatment of neuroinflammatory response.

## 2. Materials and Methods

### 2.1. Experimental Animals

Pregnant imprinting control region (ICR) (E14) mice were obtained from Coatech in Seoul, Republic of Korea. Mice were housed under constant light, temperature, and humidity conditions. All animal procedures were performed according to a protocol approved by the Yonsei University Animal Care and Use Committee, in accordance with NIH guidelines.

### 2.2. Cortical NSC Culture

Embryos (E14) were extracted from placental tissue. Cortices were aseptically dissected from the brains of fetuses and placed in Hank's balanced salt solution (HBSS) (Gibco, NY, USA). Tissues were triturated by repeated passage through a fire-polished constricted Pasteur pipette. The dispersed tissues were allowed to settle for 3 min. Supernatants were transferred to a fresh tube and centrifuged at 1,000 g for 5 min. Pellets were resuspended in NSC basal media with a proliferation supplement (Stem Cell Technologies, CA, USA), 20 ng/mL epidermal growth factor (EGF, Invitrogen, CA, USA). Cells excluding trypan blue were counted. Cells were plated in a T 75 flask at a density of 2.5 × 10^4^ cells/mL. Cultures were maintained in a humidified atmosphere of 95% air and 5% CO_2_ at 37°C. After 3 days of culture, the cells proliferated and formed primary neurospheres. The primary neurospheres composed of NSCs were harvested by centrifugation, dissociated using Accumax (Sigma, MO, USA) into single cells. The single cells were seeded in culture plates precoated with 0.001% poly L-ornithine (Sigma, MO, USA). The single cells were incubated for 5 days to form a sufficient number of neurospheres. Culture media was replaced every 3 days. NSCs of 2-3 passages were used for experiments [10].

### 2.3. Experimental Procedure

Melatonin was purchased from Sigma (Sigma, MO, USA) and dissolved in ethanol. An equivalent volume of ethanol (final: 0.01%) or distilled water was added to control wells and all melatonin-containing wells. The effects of melatonin on the proliferative activity of the NSCs were evaluated by counting the number of neurospheres and measuring the size of neurospheres. The single cell suspensions from primary neurospheres were prepared by a centrifugation (300 g, 3 min) followed by a mechanical dissociation. After incubating for 3 days, the NSCs were treated with melatonin and subsequently were cultured for 2-3 days. We used 100 nM melatonin for subsequent experiments. Also, the NSCs were exposed to 100 ng/mL or 1 *μ*g/mL LPS (Sigma, MO, USA) to study LPS-induced cytotoxic injury. After incubating for 3 days from primary neurosphere reseeding, the NSCs were pretreated with melatonin, and then after 1 day the NSCs were exposed to 100 ng/mL or 1 *μ*g/mL LPS. In the control group, cells were not exposed to LPS and melatonin. Also, NSCs were pretreated with wortmannin (a PI3K inhibitor) (Sigma, MO, USA) at 3 hr before melatonin treatment. At least three different experiments were performed using separate cell preparations, and triplicate determinations were performed for each experiment.

### 2.4. Measurement of Lactate Dehydrogenase (LDH) Activity

The release of LDH is a widely used index of cellular injury [51]. LPS-induced cytotoxicity was quantified by measuring the amount of LDH released into the culture media from injured cells [52, 53]. LDH release (cytotoxicity %) was calculated by dividing the value at the experimental time point by the maximum value. The maximum LDH release was measured after freezing each culture at −70°C overnight, followed by rapid thawing, which induced nearly complete cell damage.

### 2.5. Measurement of Nitrite Production

Nitrite production was determined in the supernatants of cultured cells. The cells were seeded in 96-well plate at density of 5 × 10^4^ cells/well. Cells were incubated overnight. Thereafter media was discarded and cells were exposed to treatments as described earlier. After treatments media from each well was transferred to fresh tube. After centrifugation, 100 *μ*L of the supernatant was transferred to fresh 96-well plate, mixed with an equal volume of Griess reagent. The plate was incubated in the dark for 15 min at room temperature. The absorbance of the reaction product was measured at 540 nm using a microplate reader (Bio-Rad, CA, USA). Nitrite concentration in control and treated cells was calculated using sodium nitrite standard reference curve and expressed as *μ*M nitrite/mL [54].

### 2.6. Measurement of Neurosphere Size

Images of the neurospheres cultures were taken using an inverted microscope (Olympus, Tokyo, Japan). The magnification of the image (×10) covered a significant area of each well from 24-well plates. An image analysis program (Image J) was used to analyze the size of neurospheres. Ten nonoverlapping fields were randomly selected from each well, and images were captured using a fluorescence microscopy (Olympus, Tokyo, Japan). Randomly chosen fields were counted. All experiments were carried out 3 times [55].

### 2.7. Neurosphere Counting

The single cell suspension of EGF-expanded NSCs was seeded in nontreatment 96-well plate (Sigma, MO, USA) at a cell density of 5 × 10^4^ cells/well and was incubated for 5 days. After treating melatonin and/or LPS, the fixed area (10 mm^2^) at the center of each well was converted into a digital image using a digital still camera (Olympus, Tokyo, Japan) and the number of neurospheres whose diameter was over 60 *μ*M was counted by Image J.

### 2.8. WST-8 Assay

The indirect counting of viable cells was carried out by WST-8 assay using a Cell Counting −8 kit (Sigma, MO, USA). The dissociated NSCs from the primary neurosphere were reexpanded with EGF in nontreatment 96-well plate (Sigma, MO, USA) at a cell density of 5 × 10^4^ cells/well. After 5-day incubation periods in the melatonin and/or LPS, 10 *μ*L of the Cell Counting −8 kit (Sigma, MO, USA) solution was added to each well and incubated for an additional 4 hr at 37°C. The absorbance at 450 nm was measured by the microplate reader (Bio-Rad, CA, USA) and the net absorbance subtracting the value of cell-free wells was calculated.

### 2.9. Hoechst-Propidium Iodide Staining

Cell viability was evaluated by staining NSCs with Hoechst 33258 dye (Sigma, MO, USA) and propidium iodide (PI; Sigma, MO, USA). Hoechst dye was added to the culture media (2-3 *μ*g/mL) and samples were maintained at 37°C for 30 min. PI solution was added (2–5 *μ*g/mL) just before observation using an Olympus microscope equipped with epifluorescence and a UV filter block. PI-positive cells were counted as dead cells.

### 2.10. Western Blot Analysis

Equal amounts of protein (50 *μ*g) were extracted from NSC cultures. They were electrophoresed on 10%–12% SDS-polyacrylamide gels. Separated proteins were electrotransferred to Immunobilon-NC membranes (Millipore, MA, USA). Membranes were blocked for 1 hr at room temperature with 5% skim milk in Tris-buffered saline and 0.1% Tween-20 (TBST). The primary antibodies used were PI3K (1 : 2000, Millipore, MA, USA), Akt (1 : 2000, Millipore, MA, USA), Nrf2 (1 : 2000, Millipore, MA, USA), and *β*-actin (1 : 1000, Santa Cruz, CA, USA). Blots were incubated with the primary antibodies overnight at 4°C. Membranes were washed three times (5 min each) with TBST. The secondary antibodies were anti-rabbit and anti-mouse (1 : 3000, New England Biolabs, MA, USA) and were incubated for 1 hr at room temperature. After washing with TBST (0.05% Tween-20) three times, immunoreactive signals were detected using chemiluminescence and an ECL detection system (Amersham Life Science, UK) with the LAS 4000 program.

### 2.11. Reverse Transcription-PCR (RT-PCR)

To confirm SOX2, TLX, and FGFR-2 expression in melatonin treated NSCs and control NSCs, reverse transcription- (RT-) PCR was performed using SOX2-specific primers and TLX-specific primers. Briefly, samples were lysed with Trizol reagent (Invitrogen, CA, USA) and total RNA was extracted according to the manufacturer's protocol. cDNA synthesis from mRNA and sample normalization were performed using RT-PCR. PCR was performed using the following thermal profile, 10 min at 95°C; 40 cycles of denaturing at 95°C for 15 seconds, annealing for 30 seconds at 60°C, and elongation at 72°C for 30 seconds; final extension for 10 min at 72°C, and paused at 4°C. PCR was carried out using the following primers: TLX F: GCTTTCTTCACAGCGGTCAC, R: GCAGACACAGCGGTCAACT; SOX2 F: CCCCCGGCGGCAATAGCA, R: TCGGCGCCGGGG AGATACAT; FGFR-2 F: ATA AGG TAC GAA ACC AGC ACT G, R: GGT TGA TGG ACC CGT ATT CAT TC; GAPDH F: GGCATGGACTGTGGTCATGAG, R: TGCACCACCAACTGCTTAGC. PCR products were electrophoresed in 1.5% agarose gels stained with ethidium bromide.

### 2.12. Immunocytochemistry

To confirm the stemness of NSCs, NSCs were plated on coverslips (5 × 10^4^ cells/well) coated with poly-D-lysine. After incubation, the medium was removed and NSCs were washed three times with phosphate-buffered saline (PBS) for immunostaining. NSCs were fixed in 4% paraformaldehyde in PBS for 30 min at room temperature and rinsed with PBS three times for 5 min and permeabilized with 0.1% Triton X-100 for 30 min at room temperature. NSCs were incubated with primary antibody overnight at 4°C. The following primary antibody was used: anti-mouse SOX2 (1 : 200, Millipore, MA, USA). After incubating the NSCs with the primary antibodies, the plates were washed three times with PBS for 5 min and were incubated with goat anti-mouse FITC-conjugated secondary antibody. NSCs were then counterstained with 4,6-diamidino-2-phenylindole (DAPI; Sigma, MO, USA) for 10 min at room temperature. Immunostained NSCs were visualized using a Carl-Zeiss confocal microscope LSM 700 (Carl-Zeiss, Jena, Germany).

### 2.13. Statistical Analysis

Statistical analyses were carried out using SPSS 18.0 software (IBM Portsmouth, IBM North Harbour, Portsmouth, Hampshire, UK). Data are expressed as the mean ± S.E.M of 3 independent experiments. Statistical significance in intergroup differences was determined by one-way analysis of variance (ANOVA), followed by Bonferroni's* post hoc* multiple comparison test. Each experiment included 3–5 repeats per condition. Differences were considered significant at ^*^ *P* < 0.05, ^**^ *P* < 0.01.

## 3. Results 

### 3.1. Melatonin Protects NSCs against LPS-Induced Inflammation

**Figure 1:**
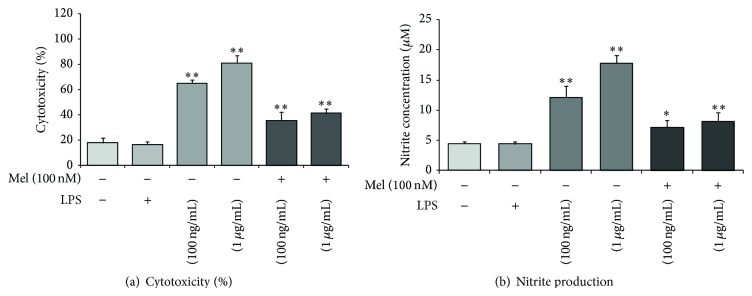
The measurement of cell cytotoxicity and nitrite production in LPS-induced inflammation. (a) Cytotoxicity (%) was measured using lactate dehydrogenase (LDH) assays. The cytotoxicity (%) was approximately 65% in the LPS (100 ng/mL) treatment group and 80% in the LPS (1 *μ*g/mL) treatment group. Upon the addition of melatonin (100 nM), the cytotoxicity (%) in all LPS treatment groups decreased 20% compared to the LPS only treatment groups. (b) Nitrite production was measured using Griess reagent assays. Nitrite concentration was approximately 13 *μ*M in the LPS (100 ng/mL) treatment group and 18 *μ*M in the LPS (1 *μ*g/mL) treatment group. Upon the addition of 100 nM melatonin, nitrite production was reduced by nearly half in all LPS treatment groups. Non: normal control, Mel (100 nM): melatonin (100 nM) treated group, LPS (100 ng/mL): LPS (100 ng/mL) treated group, LPS (1 *μ*g/mL): LPS (1 *μ*g/mL) treated group, LPS (100 ng/mL) + Mel (100 nM): melatonin (100 nM) plus LPS (100 ng/mL) treated group, and LPS (100 ng/mL) + Mel (1 *μ*g/mL): melatonin (100 nM) plus LPS (1 *μ*g/mL) treated group. Data were expressed as mean ± S.E.M and were analyzed statistically using one-way analysis of variance (ANOVA), followed by Bonferroni's* post hoc*. Each experiment included 5 repeats per condition. Differences were considered significant at ^*^ *P* < 0.05, ^**^ *P* < 0.01 (compared to the control group).

**Figure 2:**
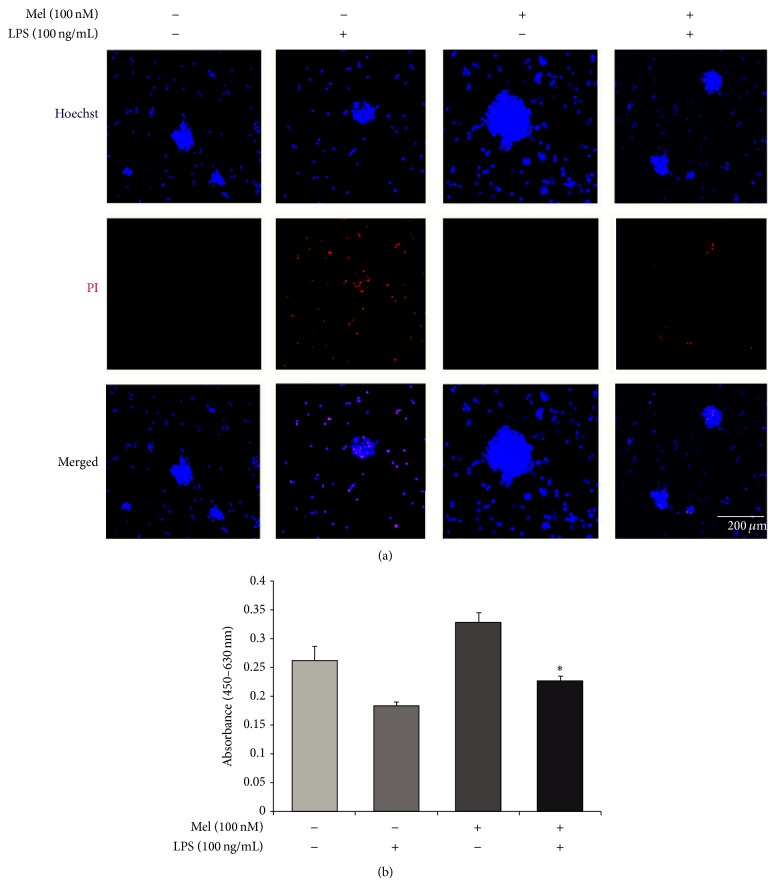
The measurement of apoptotic cells after melatonin treatment in LPS-induced inflammation. (a) Apoptotic and live cells were measured using Hoechst/PI staining. PI-positive cells (red) indicate apoptotic cells and Hoechst-positive cells (blue) indicate live cells. The LPS (100 ng/mL) treatment group had increased numbers of PI-positive cells compared to the normal control group. The melatonin (100 nM) treatment group showed decreased numbers of PI-positive cells compared to the LPS treatment group. The LPS (100 ng/mL) plus melatonin (100 nM) group had fewer PI-positive cells compared to the LPS only treatment group. Scale bar: 200 *μ*m, blue: Hoechst 33342 (Hoechst), and red: propidium iodide (PI). (b) The number of viable cells was evaluated using WST-8 assay. The melatonin treatment increased the number of viable cells compared to the normal control group and also protected the cell death under LPS-induced inflammation condition. Data were expressed as mean ± S.E.M, and each experiment included 4 repeats per condition. Differences were considered significant at ^*^ *P* < 0.05 (compared to the control group).

To check the protective effects of melatonin on NSCs in neuroinflammatory diseases, we treated LPS into NSC cultured media. Under LPS-induced inflammatory stress, melatonin attenuated apoptosis of NSC. First, to determine cytotoxicity, we conducted lactate dehydrogenase (LDH) assays. Cytotoxicity levels were approximately 65% in the LPS (100 ng/mL) treatment group and 80% in the LPS (1 *μ*g/mL) treatment group. In the melatonin (100 nM) treatment, the LPS (100 ng/mL) treatment group is decreased 20% compared to the LPS (100 ng/mL) treatment group. The LPS (1 *μ*g/mL) treatment group is decreased 35% compared to the LPS (1 *μ*g/mL) treatment group. Melatonin (100 nM) treatment attenuates cytotoxicity in LPS-induced inflammation (Figure 1(a)).  Figure 1(b) shows the nitrite concentration in all groups using Griess reagent assays. The nitrite concentration is approximately 13 *μ*M in the LPS (100 ng/mL) treatment group and 18 *μ*M in LPS (1 *μ*g/mL) treatment group. In the presence of 100 nM melatonin, LPS (100 ng/mL) attenuated NO production compared to the LPS only (100 ng/mL) treatment group. The LPS (1 *μ*g/mL) with melatonin (100 nM) treatment group decreased the nitrite concentration almost in half compared with the LPS (1 *μ*g/mL) only treatment group. In the present study, we found that melatonin inhibits NO production in LPS-treated NSCs. Melatonin may protect NSCs against LPS-induced inflammation by reducing NO. In addition, to confirm the effect of melatonin on NSC apoptosis against LPS-induced inflammatory stress, we performed Hoechst 33342 (Hoechst)/propidium iodide (PI) staining (Figure 2(a)). We investigate only the melatonin (100 nM) treatment group because Figure 1(a) shows more clear the protective effect of melatonin in the melatonin 100 nM treatment group than the melatonin 10 nM treatment group. PI-positive cells (red color) indicate apoptotic NSCs and Hoechst-positive cells (blue color) indicate live NSCs. PI-positive cells were increased in the LPS (100 ng/mL) treatment group compared to the normal control group. The melatonin (100 nM) group had fewer PI-positive cells than the LPS (100 ng/mL) treatment group. In the presence of melatonin (100 nM), the LPS (100 ng/mL) treatment group had fewer PI-positive cells compared to the LPS (100 ng/mL) only treatment group (Figure 2(a)). In addition, we measured the number of viable cells using WST-8 assay (Figure 2(b)). We confirmed that the number of viable cells was reduced under LPS-induced inflammatory condition. However, melatonin increased the number of viable cells of LPS-stimulated NSCs (Figure 2(b)).  Figure 2 suggests that melatonin inhibits the apoptosis of NSCs in LPS-induced inflammation. Taken together, these results show that melatonin protects NSCs in LPS-induced inflammation.

### 3.2. Melatonin Maintains Neurosphere Size in LPS-Treated NSCs

**Figure 3:**
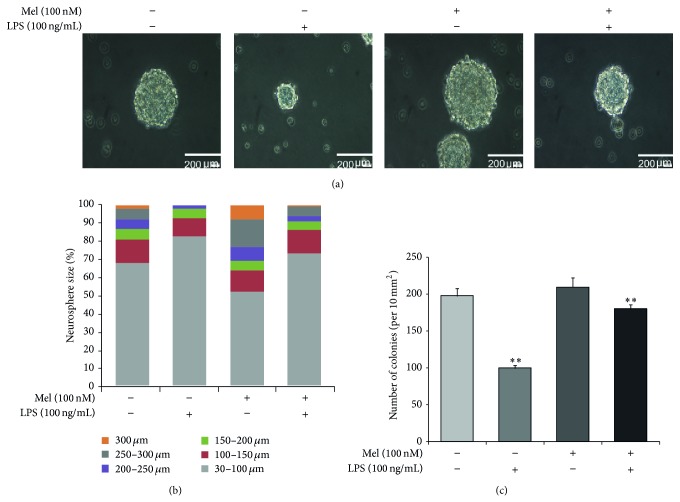
The measurement of neurosphere size in LPS-induced inflammation. (a) Neurosphere was observed using bright field microscopy and neurosphere size was measured using Image J software. Neurosphere sizes were maintained in the melatonin treatment group compared to the control group. In LPS-induced inflammation, melatonin maintained neurosphere size compared to the LPS (100 ng/mL) only treatment group. (b) The graph indicated the percentages of neurosphere size in all groups. The percentage of neurospheres greater than 300 *μ*m was higher in the melatonin treatment group compared to the normal control group. In LPS-induced inflammation, the percentage of neurospheres greater than 200 *μ*m was higher in the melatonin group compared with the LPS only treatment group. Scale bar: 200 *μ*m. (c) The number of neurospheres whose diameter was over 60 *μ*M was counted by Image J software program. The number of neurospheres was reduced under LPS-induced inflammation and was increased by melatonin treatment in spite of inflammatory condition. Data were expressed as mean ± S.E.M, and each experiment included 3 repeats per condition. Differences were considered significant at ^**^ *P* < 0.01 (compared to the control group).

We confirmed that neurosphere size was maintained by melatonin treatment in LPS-induced inflammatory conditions. Neurosphere size was measured by using bright field microscopy (Figure 3(a)). Neurosphere sizes were maintained in the melatonin (100 nM) treatment group compared to the normal control group. Additionally, melatonin (100 nM) treatment group maintains neurosphere size in LPS-induced inflammation compared to the LPS (100 ng/mL) only treatment group (Figure 3(a)).  Figure 3(b) shows that LPS (100 ng/mL) treatment decreased neurosphere size compared to the normal control group. Neurospheres greater than 200 *μ*m are fewer in the LPS (100 ng/mL) treatment group than in the normal control group. In the LPS (100 ng/mL) with melatonin (100 nM) treatment group, there are more neurospheres greater than 200 *μ*m compared to the LPS (100 ng/mL) only treatment group. In addition, we counted the number of neurospheres (Figure 3(c)). In LPS-induced inflammatory condition, the number of neurospheres was reduced compared to the normal control group. Melatonin inhibited the decrease of neurospheres in LPS-induced inflammatory condition (Figure 3(c)).  Figure 3 indicates that melatonin maintains neurosphere size in normal condition as well as in LPS-induced inflammation.

### 3.3. Melatonin Influences the Expression of SOX2, TLX, and FGFR-2 in LPS-Treated NSCs

**Figure 4:**
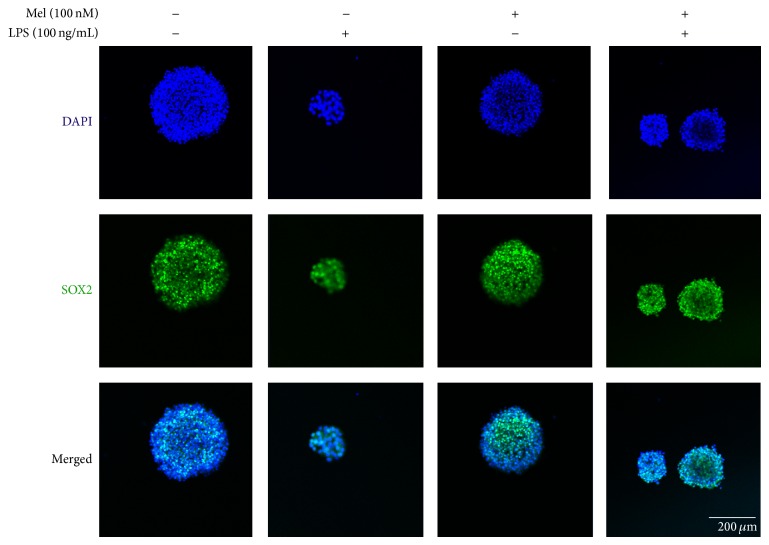
Immunofluorescent staining to check SOX2 expression. LPS-stimulated NSCs show decreased expression of SOX2 compared to the normal control NSCs. Melatonin promotes the SOX2 expression of NSCs and also melatonin slightly increases the SOX2 expression in LPS-stimulated NSCs. 4′,6-Diamidino-2-phenylindole (DAPI): blue, SOX2: green, and scale bar: 200 *μ*m.

**Figure 5:**
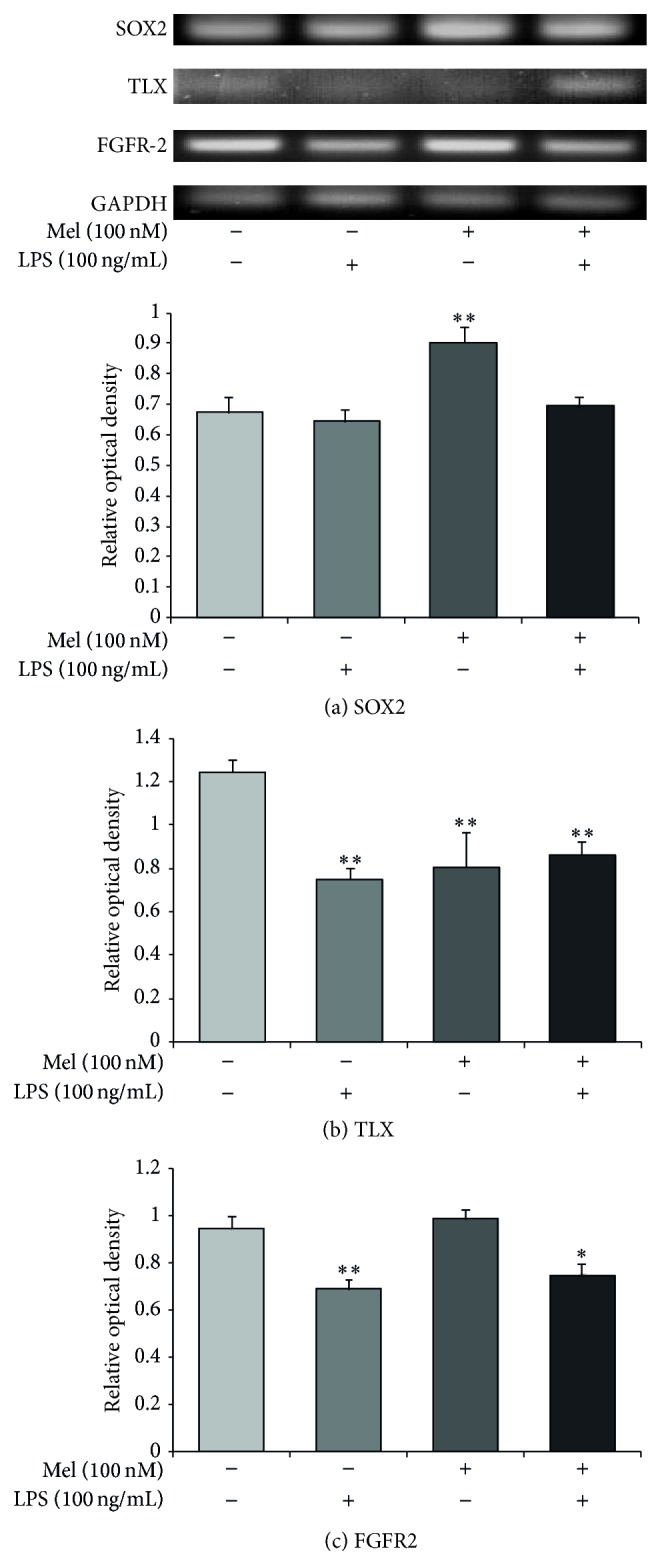
SOX2 and TLX mRNA expression after melatonin treatment in LPS-induced inflammation. (a) SOX2, (b) TLX, and (c) FGFR-2 mRNA levels were measured by using RT-PCR. The LPS (100 ng/mL) treatment group showed lower mRNA levels of SOX2, TLX, and FGFR-2 compared to the normal control group. Melatonin (100 nM) treatment resulted in higher SOX2 and FGFR-2 mRNA levels compared to the LPS (100 ng/mL) treatment group. In the LPS (100 ng/mL) plus melatonin (100 nM) treatment group, SOX2, TLX, and FGFR-2 mRNA levels were higher compared to the LPS (100 ng/mL) only treatment group. Data were expressed as mean ± S.E.M, and each experiment included 3 repeats per condition. Differences were considered significant at ^*^ *P* < 0.05, ^**^ *P* < 0.01 (compared to the control group).

To examine the expression of SOX2, we conducted immunochemical staining using SOX2 antibody in all groups (Figure 4). In the present study, we confirmed that SOX2-positive NSCs were increased by melatonin not only in normal condition but also in LPS-induced inflammatory condition (Figure 4). To measure the mRNA levels of SOX2, TLX, and FGFR-2 as markers of NSC survival and proliferation, we evaluated SOX2, TLX, and FGFR-2 using reverse transcription-PCR (RT-PCR) in all groups (Figure 5). The SOX2 mRNA level decreased in the LPS (100 ng/mL) treatment group compared to the normal control group. It increased largely by melatonin in normal condition. Also, it increased in the melatonin (100 nM) group and LPS (100 ng/mL) plus melatonin (100 nM) group compared to the LPS (100 ng/mL) treatment group (Figure 5(a)). The mRNA level of TLX decreased in the LPS (100 ng/mL) group compared to the normal control group. It slightly increased in the melatonin (100 nM) treatment group compared with the LPS (100 ng/mL) treatment group. It also slightly increased in the LPS (100 ng/mL) plus melatonin (100 nM) group compared to the LPS (100 ng/mL) treatment group (Figure 5(b)). However, we could not assure that melatonin could influence the LPS-stimulated NSCs because LX mRNA level in melatonin treatment group showed significant increase of TLX mRNA level in spite of the LPS-induced inflammation compared to the LPS treatment group. The mRNA level of FGFR-2 decreased in the LPS (100 ng/mL) treatment group compared to the normal control group. Also, it increased by melatonin under LPS-induced inflammatory condition compared to the only LPS treatment group (Figure 5(c)). The pattern of FGFR-2 mRNA level shows that melatonin may promote the expression of FGFR-2 mRNA in LPS-induced inflammatory condition. Figure 5 indicates that melatonin (100 nM) may enhance the mRNA expression of SOX2 and FGFR-2 both in normal condition and in LPS-induced inflammation. In the present study, our results support that melatonin may affect SOX2, TLX, and FGFR-2 expression in LPS-treated NSCs and suggest that melatonin could regulate NSC's proliferation and survival in neuroinflammatory disease.

### 3.4. Melatonin Activates the PI3K/Akt/Nrf2 Signaling in LPS-Treated NSCs

**Figure 6:**
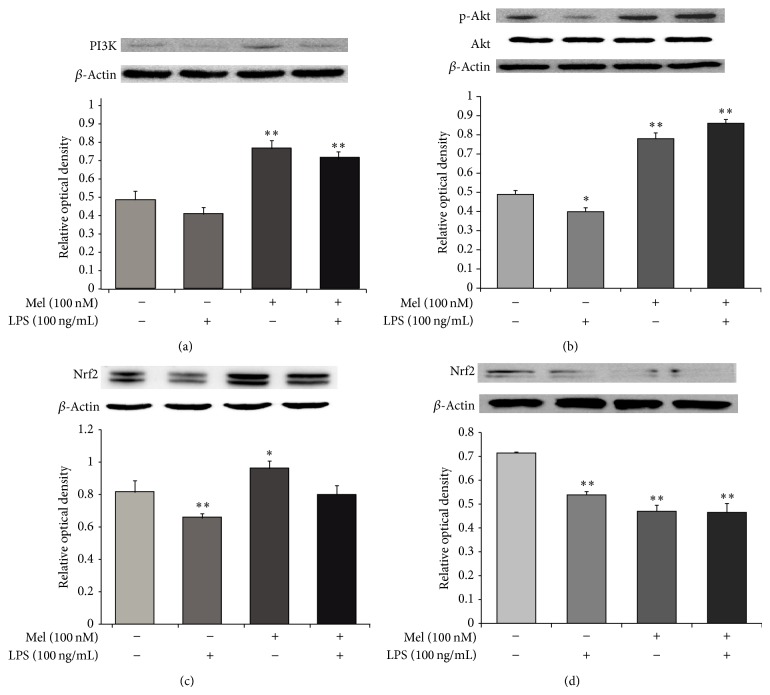
The measurement of PI3K/Akt/Nrf2 signaling after melatonin treatment in LPS-induced inflammation. (a) Western blotting experiments showed that the relative protein expression of PI3K decreased in the LPS (100 ng/mL) treatment group compared to the normal control group. The relative level of PI3K was elevated in the LPS (100 ng/mL) plus melatonin (100 nM) treatment group compared to the LPS only treatment group. (b) Western blot analyses showed that the relative protein level of Akt decreased in the LPS (100 ng/mL) treatment group compared to the normal control group. The relative level of Akt was elevated in the LPS (100 ng/mL) plus melatonin (100 nM) treatment group compared to the LPS only treatment group. (c) Western blot analyses showed that the relative protein level of Nrf2 decreased in the LPS (100 ng/mL) treatment group compared to the normal control group. The relative level of Nrf2 was elevated in the LPS (100 ng/mL) plus melatonin (100 nM) treatment group compared to the LPS only treatment group. (d) Western blot analyses showed that the relative protein level of Nrf2 decreased in the melatonin (100 nM) treatment group compared to LPS (100 ng/mL) treatment group. Also, the relative level of Nrf2 was also attenuated in the LPS (100 ng/mL) plus melatonin (100 nM) treatment group. In all groups, NSCs were treated with 100 nM wortmannin (a PI3K inhibitor) at 3 hr before melatonin or/and LPS treatment. *β*-Actin was used as an internal control. Data were expressed as mean ± S.E.M, and each experiment included 4 repeats per condition. Differences were considered significant at ^*^ *P* < 0.05, ^**^ *P* < 0.01 (compared to the control group).

To measure the protein expression of PI3K, Akt, and Nrf2, we performed western blot analyses (Figure 6).  Figure 6(a) shows that the expression of PI3K decreased during LPS-induced inflammation. However, expression of PI3K increased in the melatonin (100 nM) group and the LPS (100 ng/mL) plus melatonin (100 nM) treatment group.  Figure 6(b) shows that Akt expression decreased during LPS-induced inflammation. Akt expression increased in the melatonin (100 nM) group and the LPS (100 ng/mL) plus melatonin (100 nM) treatment group.  Figure 6(c) shows that Nrf2 expression decreased in LPS-induced inflammation. However, the expression of Nrf2 increased in the melatonin (100 nM) group and the LPS (100 ng/mL) plus melatonin (100 nM) treatment group. To determine the upstream signaling pathways responsible for upregulation of Nrf2 expression, we examined the effect of melatonin on the phosphorylation of Akt. Wortmannin (a PI3K inhibitor) suppressed melatonin-induced activation of Akt (Figure 6(d)). We examined the effects of wortmannin on Nrf2 expression. These data suggest that PI3K and Akt are involved in melatonin-induced upregulation of Nrf2 in NSCs. Taken together, we suggest that melatonin may promote activation of PI3K/Akt/Nrf2 signaling and also may influence the survival of NSCs under LPS-induced inflammation.

## 4. Discussion

Melatonin is a potent free radical scavenger capable of preventing oxidative stress in a number of biological systems [56–58]. Melatonin has important actions in oxidative defense by stimulating antioxidative enzymes [59]. NSC has the pluripotential ability and so has been known as a therapeutic target to improve the recovery of injured tissue in neuroinflammatory disease [60]. NSC's survival is important to enhance the therapeutic effect at injury site [61]. In the present study, we investigated the protective effect of melatonin in LPS-treated NSCs* in vitro*. NO can induce apoptosis in various cells, including neurons [31, 32], and contributes to the death of neurons in CNS disorders [33, 34]. Also, NO is related to NPC survival and cell fate determination of NPCs [35]. Tanaka et al. demonstrated the role of NO in neural proliferation [36]. NO regulates both termination of proliferation and initiation of differentiation of NSCs in the developing cortex [62]. In isolated NSCs from the subventricular zone (SVZ), high NO concentrations inhibit NSC proliferation and promote differentiation of precursors into astrocytes [63, 64]. Vilar et al. demonstrated that melatonin suppresses NO production in glial cultures by proinflammatory cytokines through p38 MAPK inhibition [37]. In the present study, our data shows the relationship between melatonin and NO in LPS-treated NSCs. In addition, several studies demonstrated that melatonin is linked with NSC proliferation and the neurosphere formation [10, 65]. In the present study, we confirmed that melatonin is associated with neurosphere formation and maintains the neurosphere during LPS-induced inflammation. In the CNS, SOX2 as a member of the Sox family of transcription factors is expressed in NSCs from neurogenic regions and regulates stem cell proliferation and differentiation [66]. SOX2 maintains the NSC state by controlling proliferation and differentiation [66–68]. Additionally, SOX2 promotes self-renewal potential and inhibits apoptosis and differentiation [41, 69, 70]. The orphan nuclear receptor TLX is an essential regulator of NSC self-renewal. TLX maintains adult NSCs in an undifferentiated and self-renewable state [42]. In adult brain, TLX-positive cells in the hippocampal dentate gyrus play an important role in learning and memory [71]. TLX regulates adult NSC self-renewal [42] through transcriptional repression of downstream target genes by binding with histone-modifying enzymes [72–74] or by activating the Wnt/*β*-catenin pathway [75]. FGF-2 is associated with proliferating NSCs* in vivo* and regulating NSCs self-renewal* in vitro* [76–79]. In the present study, our data suggests that melatonin influences the expression of SOX2, TLX, and FGFR-2 in LPS-treated NSCs. Nrf2 is a transcriptional activator of cytoprotective genes. It activates transcription in response to ROS [80, 81]. Under oxidative stress, Nrf2 translocates to the nucleus where it binds DNA promoters and initiates transcription of antioxidative genes and their proteins [82, 83]. Nrf2 activates a cellular rescue pathway that protects against the LPS-induced inflammatory response [47]. Nrf2 enhances cytoprotection by activating PI3K-Akt signaling [84, 85]. Several studies demonstrated that pharmacological inhibition of the PI3K-Akt pathway represses nuclear translocation of Nrf2 [86, 87]. Negi et al. [88] reported that melatonin ameliorates neuroinflammation and oxidative stress via Nrf2 activation. Nrf2 upregulation by melatonin resulted in increased expression of the antioxidant enzyme heme oxygenase-1 (HO-1) [88]. HO-1 is the rate-limiting enzyme that catalyzes heme to biliverdin, carbon monoxide (CO), and free iron. The byproducts of HO-1 catabolism have been shown to exhibit protective effects against oxidative and inflammatory stimuli [89]. HO-1 expression also is related to PI3K/Akt pathway activation [85, 90, 91] to protect cells from oxidative damage and cerebral ischemia* in vitro* and* in vivo* [92, 93]. Several studies demonstrated that melatonin increases the mRNA and protein levels of antioxidant enzymes via Nrf2 activation [49, 50]. In the present study, we confirmed that melatonin is related to Nrf2 activation in LPS-treated NSCs. Our consequences indicate that melatonin may promote antioxidant gene expression by regulating Nrf2 activation to protect NSCs under LPS-induced inflammation. In addition, considering that NSCs regulate the survival and neurogenesis by PI3K/Akt signaling [94], our results indicate that melatonin may promote NSC survival by regulating Nrf2 activation under LPS-induced inflammation. To conclude, this study suggests five points: (1) melatonin inhibits NO production in LPS-treated NSC, (2) melatonin attenuates the apoptosis of NSC in LPS-induced inflammation, (3) melatonin affects the neurosphere formation of NSC against LPS-induced inflammation, (4) melatonin may regulate SOX2 and FGFR-2 expression in LPS-treated NSC, and (5) melatonin may induce the activation of Nrf2 through PI3K/Akt signaling pathway in LPS-treated NSC. Hence, we suggest that melatonin may influence the survival of NSCs in neuroinflammatory diseases.
